# Quantitative RT-PCR Gene Evaluation and RNA Interference in the Brown Marmorated Stink Bug

**DOI:** 10.1371/journal.pone.0152730

**Published:** 2016-05-04

**Authors:** Raman Bansal, Priyanka Mittapelly, Yuting Chen, Praveen Mamidala, Chaoyang Zhao, Andy Michel

**Affiliations:** 1 Department of Entomology, Ohio Agricultural Research and Development Center, The Ohio State University, Wooster, OH, 44691, United States of America; 2 Department of Biotechnology, University College of Science, Telangana University, Dichpally, Nizamabad, Telangana, 503322, India; United States Department of Agriculture, Beltsville Agricultural Research Center, UNITED STATES

## Abstract

The brown marmorated stink bug (*Halyomorpha halys*) has emerged as one of the most important invasive insect pests in the United States. Functional genomics in *H*. *halys* remains unexplored as molecular resources in this insect have recently been developed. To facilitate functional genomics research, we evaluated ten common insect housekeeping genes (*RPS26*, *EF1A*, *FAU*, *UBE4A*, *ARL2*, *ARP8*, *GUS*, *TBP*, *TIF6* and *RPL9*) for their stability across various treatments in *H*. *halys*. Our treatments included two biotic factors (tissues and developmental stages) and two stress treatments (RNAi injection and starvation). Reference gene stability was determined using three software algorithms (geNorm, NormFinder, BestKeeper) and a web-based tool (RefFinder). The qRT-PCR results indicated *ARP8* and *UBE4A* exhibit the most stable expression across tissues and developmental stages, *ARL2* and *FAU* for dsRNA treatment and *TBP* and *UBE4A* for starvation treatment. Following the dsRNA treatment, all genes except *GUS* showed relatively stable expression. To demonstrate the utility of validated reference genes in accurate gene expression analysis and to explore gene silencing in *H*. *halys*, we performed RNAi by administering dsRNA of target gene (catalase) through microinjection. A successful RNAi response with over 90% reduction in expression of target gene was observed.

## Introduction

Determining gene function within a genome (i.e. functional genomics) provides insight into the molecular interactions between organisms and their environments. Insects present good test cases for functional genomics because of their fast generation time, reproductive output, ease of rearing large populations, and a wealth of genomic knowledge. Two techniques are needed for successful functional genomics: accurate quantification of gene expression and assessment of gene function by silencing gene expression.

Real-time reverse transcription-PCR (qRT-PCR) has emerged as a powerful tool to measure gene expression [1]. qRT-PCR is preferred over the traditional gene expression measurements such as northern blot analysis, competitive RT-PCR, *in situ* hybridization or RNase protection assay because it is highly accurate, less labor-intensive, less time-consuming (no post-PCR processing), provides higher resolution, and, most importantly, is quantitative [2,3]. Furthermore, qRT-PCR is highly reproducible and sensitive due to its ability to detect transcripts expressed at low levels [3–5].

Many factors influence the accuracy and interpretation of qRT-PCR, including the quantity and quality of the starting material, RNA extraction, cDNA synthesis, and other laboratory procedures. To limit variability, normalization of the data occurs by comparing target gene expression levels to that of reference genes. Also known as housekeeping genes (HKG), reference genes are assumed to have stable expression across various biotic and abiotic stresses, and treatments (*e*.*g*. tissues, and developmental stages) [6]. Depending on the experiment, these assumptions may not be valid; indeed recent research shows that a condition-specific reference gene for a given species needs to be identified for accurate measurements of gene expression [3].

To evaluate gene silencing, measuring gene expression is critical, as it validates decreased expression of the target gene. One of the most common ways to silence gene expression is through RNA-interference (RNAi). RNAi results in a sequence specific knockdown of gene expression at the post-transcriptional level, as introduced dsRNA causes the degradation of identical mRNAs [7]. The dsRNA can be introduced in different ways, but is most commonly injected into an organism. However, both the mechanical stress (due to needle injection) and presence of exogenous dsRNA may induce changes in HKG expression and alter the subsequent validation of target-gene silencing through qRT-PCR. Thus, identification and validation of HKG expression stability is a pre-requisite for developing RNAi in any organism.

In recent years, the brown marmorated stink bug (*Halyomorpha halys*) has emerged as one of the most important insect pests in United States [8]. *H*. *halys* is a native of Asia and is assumed to have invaded North America in the 1990s, as it was first observed in the United States in Allentown, PA around 1996 [9]. Since 1996, *H*. *halys* has spread rapidly across North America and, as of October 2015, it had been detected in 42 U.S. states and two Canadian provinces (www.stopbmsb.org). *H*. *halys* causes significant economic losses on tree fruits, ornamentals, vegetables, and field crops [8]. The mid-Atlantic region of the U.S. experiences the most damage, where severe losses to apples, peaches, sweet corn, peppers, tomatoes, and soybean occurred in 2010 [8]. The estimated damage to apples alone in this area exceeded $37 million [10]. Insecticide applications have increased four-fold to prevent *H*. *halys* damage [11]. However, natural enemies are also impacted by broad spectrum insecticides such as pyrethroids, causing an emergence of secondary pests such aphids, scales and mites [8]. Given the high costs and off-target impacts of insecticides, novel measures such as those targeting molecular physiology are needed to manage *H*. *halys*. But, molecular physiology of *H*. *halys* has not been explored due to recent generation of molecular resources and the subsequent lack of functional genomics research.

Here, our goals were to determine the best reference genes for precise quantification of mRNA transcripts in *H*. *halys*, and to explore gene silencing through RNAi technique in this insect. We evaluated ten, common insect HKGs for their stability in *H*. *halys* among various treatments, including different tissues and development stages, starvation stress, and dsRNA injection. We demonstrated the utility of validated reference genes in accurate expression analysis during the exploration for successful gene silencing in *H*. *halys* by dsRNA injection. Overall, our data provided recommendations as to which HKGs should serve as the reference genes in qRT-PCR and functional genomic experiments with *H*. *halys*.

## Methods

### Selection of HKGs

Based on information available on commonly used insect reference genes in the literature, we selected ten *H*. *halys* transcripts to evaluate as candidate reference genes (Table 1). We identified these transcripts from a *H*. *halys de novo* assembly having 79,855 high-quality transcripts. The *de novo* assembly originated from eight RNA-Seq libraries, each corresponding to an individual *H*. *halys* adult, prepared using the TruSeq RNA Sample Preparation Kit (Illumina Inc., San Diego, CA, US), following the manufacturers protocol and sequenced on HiSeq 2000 flow cell (Illumina Inc., San Diego, CA, US). More details on library construction, sequencing, and data analysis are provided elsewhere (Bansal and Michel, Submitted). Homology searches for transcript sequences were performed using Blast2GO software [E-value cut-off 10^−3^] [12–14]. All sequence data were deposited in the GenBank under the BioProject accession PRJNA263732. The mRNA sequences for ten candidate reference genes tested in this study are provided in the S1 Appendix.

**Table 1:** Description of candidate reference genes for qRT-PCR studies in brown marmorated stink bug.

Gene symbol*	*Tribolium castaneum* homolog locus	Identity(%)	E value	*T*. *castaneum* locus description	Function
*RPS26*	XP_973916.1	87	3e-63	40S ribosomal protein S26	Structural constituent of 40S ribosomal unit
*EF1A*	XP_966355.1	95	0.0	Elongation factor-1-alpha	Translational elongation
*FAU*	XP_971838.1	64	2e-40	Ubiquitin-like protein FUBI	Unknown
*UBE4A*	XP_966451.1	42	0.0	Ubiquitin conjugation factor E4	Binds ubiquitin duringprotein recycling
*ARL2*	XP_001808435.1	86	4e-109	ADP-ribosylation factor 2 A	Regulator of vesicular traffic and actin remodeling
*ARP8*	XP_008199828.1	50	0.0	Actin-related protein 8	Chromatin remodeling
*GUS*	XP_969423.1	61	0.0	β-glucuronidase	Catalyze the hydrolysis of oligosaccharides
*TBP*	XP_966659.1	50	0.0	TATA-binding protein-associated factor 172	Transcription factor
*TIF6*	XP_970112.1	81	8e-141	Eukaryotic translation initiation factor 6	Translation initiation
*RPL9*	XP_974780.1	86	3e-120	60S ribosomal protein L9	Structural constituent of 60S ribosomal unit

*The transcript sequences for genes described in this study are provided in S1 Appendix.

### *H*. *halys* laboratory rearing

Our laboratory colony originated from adult *H*. *halys* collected in a soybean field at the Ohio Agricultural Research and Development Center (OARDC; 40° 45' 52'' N, 81° 54' 34''W, Wooster, OH, USA) during August and September 2012. In our colony, *H*. *halys* individuals are kept in rearing cages (299 cm cube with 24 x 24 mesh; BioQuip Products, Rancho Dominguez, CA) and stored in a growth chamber [28±2°C, 60–70% relative humidity and 16:8 (light:dark) photoperiod]. *H*. *halys* individuals feed on a mixed diet of corn cobs, green beans, grapes, lettuce and carrots. Additional standard practices for rearing *H*. *halys*, as described by [15], were followed. To maintain genetic diversity, the laboratory colony is augmented with *H*. *halys* from local homes or fields (within Wooster) annually.

### *H*. *halys* tissues and developmental stages

We chose to compare HKG expression in several important tissues: gut, salivary gland, fat body, malpighian tubule, and ovary. We dissected *H*. *halys* females in phosphate-buffered saline, pH 8.0 under a dissecting microscope. Different tissues from 6 adults were pooled to constitute one biological replicate, with three biological replicates for each tissue. *H*. *halys* has 5 nymphal stages, and all five stages plus the adult were collected from the laboratory colony to determine the expression of HKG in different developmental stages. A pool of 5 insects from each developmental stage constituted one biological replicate and there were three biological replicates for each developmental stage.

### Starvation and feeding treatments

Five-day old *H*. *halys* adults (both male and female) were starved for 24 h. To avoid cannibalism, insects were kept individually in 50 ml Falcon^®^ tubes, with holes in the lid for air circulation. For the alternative treatment, adult *H*. *halys* fed on the same mixed diet as was previously described. We dissected starved and fed adults in phosphate-buffered saline (pH 8.0) to obtain the gut tissues. Before multiple washings with buffer, we punctured the gut to remove lumen contents. Following dissection, the tissues were stored at -80°C until RNA was isolated. Gut tissues from 6 adults (3 males and 3 females) were pooled to constitute one biological replicate and there were three biological replicates each for fed and starved *H*. *halys*.

### dsRNA synthesis and injections

To explore RNAi in *H*. *halys*, we selected *Catalase* (*HhCAT*) as the target gene. Catalase, in general is an antioxidant enzyme that protects against oxidative stress by converting hydrogen peroxide to water and oxygen [16]. From the *H*. *halys* transcriptome (Bansal and Michel, Submitted), a 1,529 bases transcript encoding a protein showing strong similarity to insect catalases was selected. On searching the two publically available *H*. *halys* transcriptomes [17,18], we found one corresponding mRNA sequence (encoding catalase) in each transcriptome. The *HhCAT* transcript from our database was nearly identical to the ones found in publically available transcriptomes: identities 1,528/1,529 to putative catalase having accession# GBHT01002016 in [17] and identities 1,527/1,529 to putative catalase having accession# GDCO01082203 in [18]. At the mRNA and protein level, three *H*. *halys* catalase transcripts in different transcriptome datasets appeared to be transcribed from the same gene; however genome scanning is needed for the confirmation. The *HhCAT*’s transcript sequence is provided in S1 Appendix.

For dsRNA synthesis, initially a 704bp fragment was amplified from the first strand cDNA preparation derived from total RNA of *H*. *halys*. The sense and antisense primer sequences used were AAGACAGCGCAAGGAGAAAG and GATGCCCTGCGAAGATGATT, respectively. For control, the green fluorescent protein (GFP)-fragment was amplified from a plasmid containing a GFP insert. Both amplified fragments were purified using QIAprep Spin Miniprep Kit (Qiagen, Germantown, MD). The dsRNAs were synthesized using MEGAscript RNAi kit (Life Technologies Corporation, Carlsbad, CA) following the manufacturer’s protocol. The dsRNA purity was checked on agarose gel electrophoresis. The dsRNA was quantitated using Nanodrop 2000c (Thermo Scientific, Hudson, NH) spectrophotometer. Before injections, insects were anesthetized on ice. We injected 2^nd^ instar nymphs for HKG evaluation and adults for silencing *HhCAT*. For *HhCAT* expression silencing, we used 3 replications of 22 individuals each (66 total individuals for both *HhCAT* and the GFP control). A total of 500ng dsRNA was injected into each insect with 414 nL nuclease free water (6 separate injections of 69 nL each), using a micro-injector (Nanoject II, Drummond Scientific Company, Broomall, PA). The injection site was the ventral metathoracic region near the hind leg coxa. After injections, stink bugs were rested for 1 hour before being moved to rearing cages for observation. For molecular analysis, *H*. *halys* nymphs were collected at 48 h post injection whereas adults were collected at 2, 4, and 6 days after injections (DAI). At each time point, 2 male and 2 female *H*. *halys* adults were collected and stored at -80°C until further processing. To measure mortality due to *HhCAT* expression silencing, we compared survival among dsGFP and dsCAT injected individuals using 30 adults (66 total minus the 36 removed for measuring gene expression). We chose this experimental design as opposed to two different groups (one for mortality and one for gene expression) based on space limitations and for preliminarily analysis of *HhCAT* expression silencing. Statistical differences in mortality were evaluated using t-test with average mortalities. Cumulative mortality was compared using Kaplan-Meier survival curves and the log-rank test [19].

### RNA extraction, cDNA synthesis, and qRT-PCR analysis

Frozen samples from each treatment were processed for total RNA extraction using the PureLink^®^ RNA Mini Kit (Life Technologies Corporation, Carlsbad, CA), as per the manufacturer’s protocol. To remove DNA contamination, samples were treated with PureLink^®^ DNase (Life Technologies Corporation, Carlsbad, CA, US). RNA quality was checked using a Nanodrop 2000c (Thermo Scientific, Hudson, NH, US). The first-strand cDNA was prepared using iScript^™^ advanced cDNA synthesis kit (Bio-Rad Laboratories, Hercules, CA, USA).

Specific primers for each HKG were designed using Beacon Designer version 7.0 (Premier Biosoft, Palo Alto, CA) (Table 1). The sense and antisense primer sequences for qRT-PCR analysis during the *HhCAT* RNAi experiment were CTTCGACAGGGAGAGGAT and CTGGGTGATGTCGTTAGTG, respectively. The qRT-PCR reactions were performed with iQ SYBR green super mix on a CFX-96 thermocycler system (Bio-Rad, Hercules, CA, USA). Each qRT-PCR reaction was performed with 2 μl (100 ng/μl) of cDNA template, 0.5 μl (100 μM) of each primer and 5 μl of iQ SYBR green super mix (Bio-Rad, Hercules, CA, USA) in 10 μl total volume. Each reaction was done in duplicate, in a 96-well optical-grade PCR plate and sealed with optical sealing tape (Bio-Rad Laboratories, Hercules, CA). PCR amplifications included the following cycling conditions: one cycle at 95°C (3 min), followed by 40 cycles of denaturation at 95°C (30 seconds), annealing and extension at 55°C for 45 sec. Finally, melt curve analyses occurred by slowly heating the PCR mixtures from 65°C to 95°C in increments of 0.5°C every 5 s with simultaneous measurements of the SYBR green signal intensities. Amplification efficiencies (E) and correlation coefficients (R^2^) for each primer pair were calculated as described in Bio-rad’s *Real-Time PCR Applications Guide* (catalog #170–9799). Relative expression values of genes in biological samples were calculated using the Ct method [20].

### Stability analysis of candidate reference genes

Four algorithms were used to estimate the stability of HKG and determine their suitability as reference genes: GeNorm [21], Normfinder [22], BestKeeper [23] and RefFinder (www.leonxie.com/referencegene.php). GeNorm calculated the ‘M’ value, with a lower value for ‘M’ indicating a more stable expression or lower variation [21]. ‘M’ is calculated by a geometric averaging of the mean pairwise variation of a HKG to all the other HKGs. HKGs showing high ‘M’ values (M>1.5) are not considered for normalization studies. NormFinder determines the expression stability by considering intra- and inter group variations for candidate reference genes [22]. NormFinder provides the stability value for each HKG, which is a direct measure of the estimated expression variation and enables standard errors to be including during normalization [22]. The BestKeeper program determines the stability of a HKG based on the standard deviation (SD) of the Ct values [23]; the lower the SD, the better the HKG is as a reference. RefFinder is a comprehensive tool which integrates the output from geNorm, Normfinder, BestKeeper, and the comparative Ct method [24] and then ranks the HKGs based on stability. RefFinder allocates an appropriate weight to each gene on the basis of its ranking in each program and then calculates an overall ranking from the geometric mean of those weights. BestKeeper and RefFinder used raw Ct values, whereas GeNorm and Normfinder used expression values calculated as 2^(-ΔCt)^.

## Results

### Optimization of qRT-PCR assay for candidate genes

One of the first steps for reference gene suitability is specific PCR amplification. Initially, we tested primer specificity for each HKG by reverse transcription polymerase chain reaction (RT-PCR). The PCR amplifications for each primer pair yielded a single-specific band of expected size after agarose electrophoresis (S1 Fig). Further, the melting curve analysis in the qRT-PCR reaction showed a single peak for each primer pair, suggesting the absence of any non-specific amplification (S2 Fig). A standard curve was generated for each gene using a serial dilution of the pooled cDNAs from each treatment. The standard curve for HKG provided the amplification efficiency and correlation coefficient, which are shown in Table 2. The correlation coefficients (R^2^) for all primer pairs ranged between 0.92 and 0.99. Amplification efficiency was consistently high for all primers except for two HKGs: eukaryotic translation initiation factor 6 (*TIF6–*82.66%) and 60S ribosomal protein L9 (*RPL9–*82.57%). Consequently, due to their relatively lower amplification efficiencies, we did not include *TIF6* and *RPL9* in further analyses.

**Table 2:** Primer sequences and amplicon characteristics of candidate reference genes for qRT-PCR studies in brown marmorated stink bug.

Gene symbol	Primer sequences	Amplicon Length (bp)	Product Tm (°C)	Amplification efficiency E (%)	Correlation coefficient (R^2^)
*RPS26*	CCTACCAAAGCCTTCTGAATATACCGTAATTGCCATAAGAG	79	79.0	94.98	0.92
*EF1A*	GCTGATTGTGCTGTGTTAACGAGTCTGTCCATTCTT	78	79.0	104.47	0.99
*FAU*	GGAACTGTGAGGTCAAGATTAGCAGCATCAGGAACT	80	76.5	85.58	0.98
*UBE4A*	CGACCATCCTTAGAGACAGATTACTGCCATGCTCAA	183	78.5	98.39	0.96
*ARL2*	AGTTCTCGTGACTAATCGCTTGAATACCTGTCCAGTA	127	76.5	86.31	0.92
*ARP8*	CCAACAATCAGCGAAGGTATGAGCAACTACTTCAGGAATAACTCT	80	76.5	103.42	0.95
*GUS*	ATCGTATCAGCACCGTATTTGAAGCAGAAGCAGAAC	76	79.0	112.60	0.93
*TBP*	GTATATTGGTGGAAATGAAGATAAGTATCCAAGCAT	97	75.0	95.98	0.93
*TIF6*	CACGCATTGTTGTAGTAATGCTGGTATGGTAGTGAAT	130	78.5	82.66	0.92
*RPL9*	CGGAGACATAAAGACCATAAGGACGAACTTATTATTGAG	127	78.5	82.57	0.99

### Expression profiles of candidate reference genes

**Fig 1:**
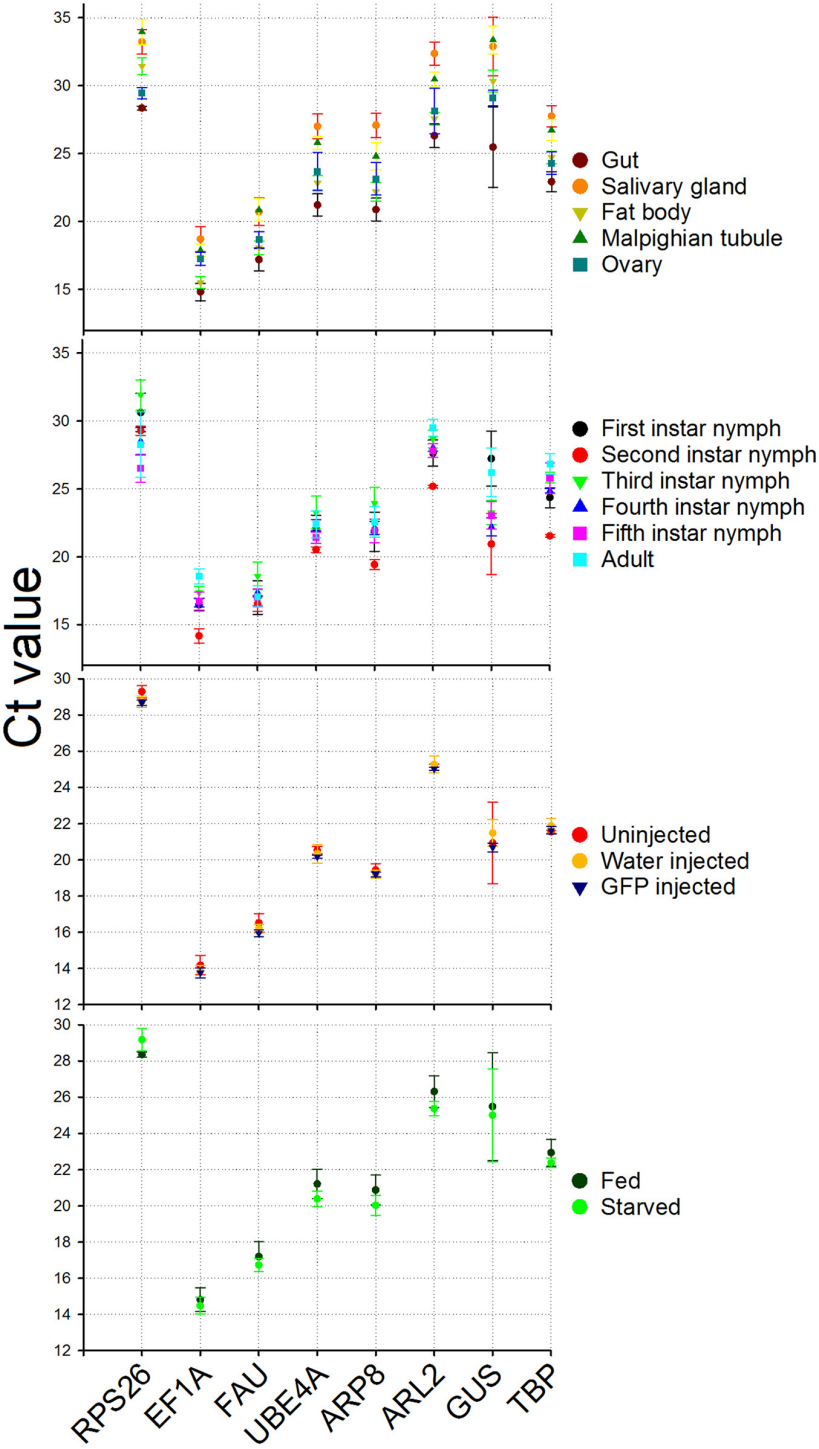
Ct values±S.D. (standard deviation) obtained for candidate reference genes in brown marmorated stink bug under different experimental conditions. Each data point represents the Mean±S.D. of Ct values for three biological replications in each treatment. Details on candidate reference genes are provided in Table 1.

Profiling HKG expression provided an overall representation of the variability for each treatment (Fig 1). The expression for *EF1A* was consistently higher compared to other genes in all treatments, with mean Ct value ranging between 13 and 19. *RPS26* was the least expressed gene with mean Ct value ranging between 26 and 32. The experimental treatment did influence the degree of variability in HKG expression. For example, *ARL2* had less variation (below 1 cycle) in RNAi injection when compared to expression among tissues (nearly 7 cycles greater). Across all treatments, a few genes showed relatively smaller variation (nearly 5 cycles) in their expression (*e*.*g*. *FAU* at nearly 5 cycles), while others had higher variation in expression (*e*.*g*. *GUS* at nearly 14 cycles). Though all HKG exhibited expression variability across all treatments, in general, their expression seemed to be relatively less influenced by injection (Fig 1).

### geNorm analysis

**Fig 2:**
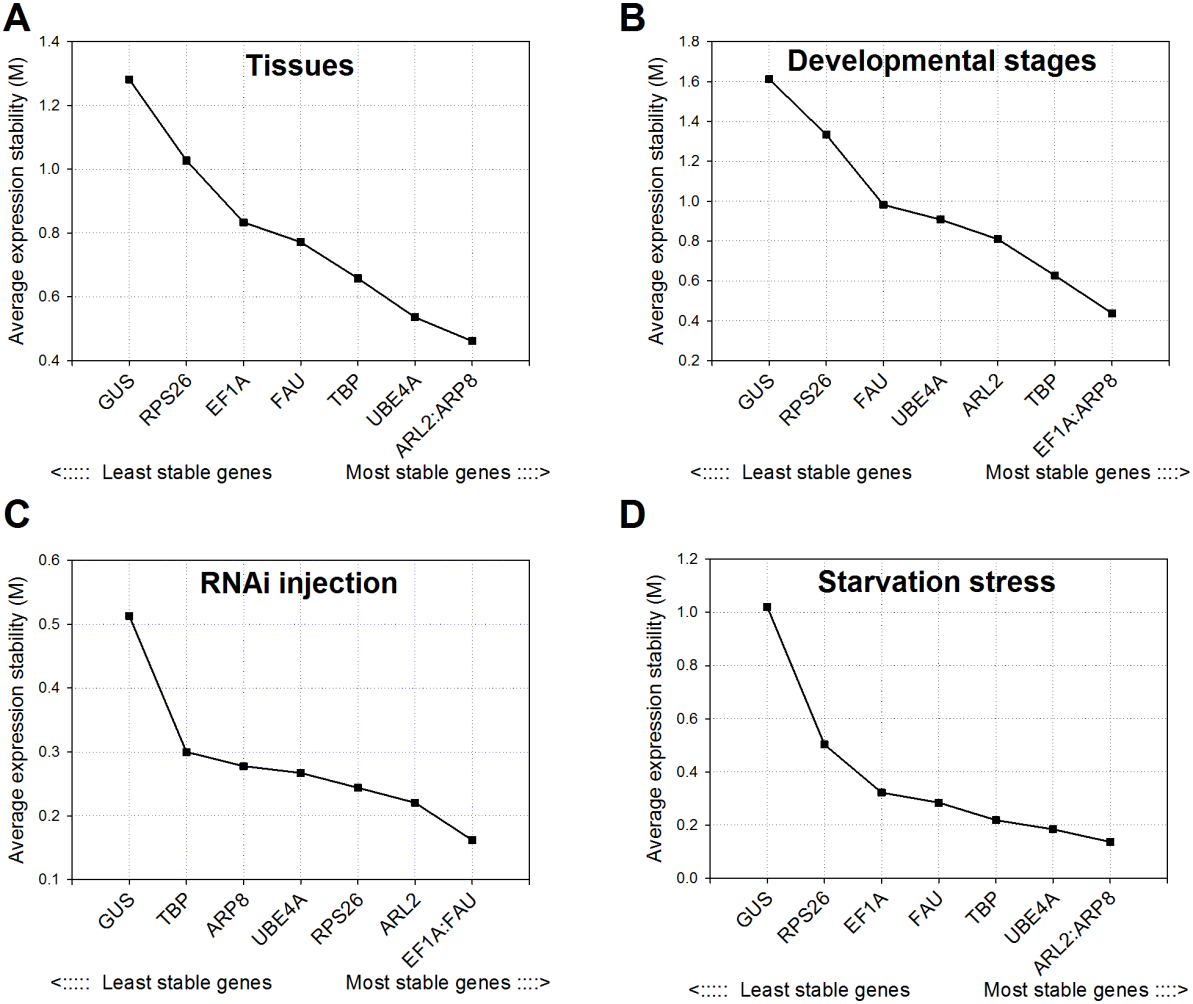
Average expression stability (M) and ranking of candidate reference genes in brown marmorated stink bug as calculated by geNorm. M values and rankings are presented for brown marmorated stink bug under different experimental conditions as indicated in each figure. Details on candidate reference genes and primer sequences are provided in Tables 1 and 2, respectively.

Among the tissues, geNorm calculated the lowest average expression stability value (M) for *ARL2* and *ARP8* (0.4613) and the highest for *GUS* (1.2817), suggesting that *ARL2* and *ARP8* had the most stable expression and that *GUS* had the least stable expression (Fig 2A). *ARP8* was also found to be the most stable along with *EF1A* (M value 0.4380) across developmental stages. Following the injections in *H*. *halys*, *EF1A* and *FAU* were the most stable genes (M value 0.1620). In fact, the M value of all HKG, except *GUS*, showed minor variation and remained lower than 0.3000 (Fig 2C) in RNAi injected treatments. Starvation stress revealed a similar pattern as different tissues, with *ARL2* and *ARP8* as most stable genes (M value 0.1368) (Fig 2D). *GUS* remained the least stable gene consistently in all treatments (Fig 2A–2D), and exceeded the 1.5 threshold of reference gene suitability in developmental stages (1.612).

### NormFinder analysis

**Fig 3:**
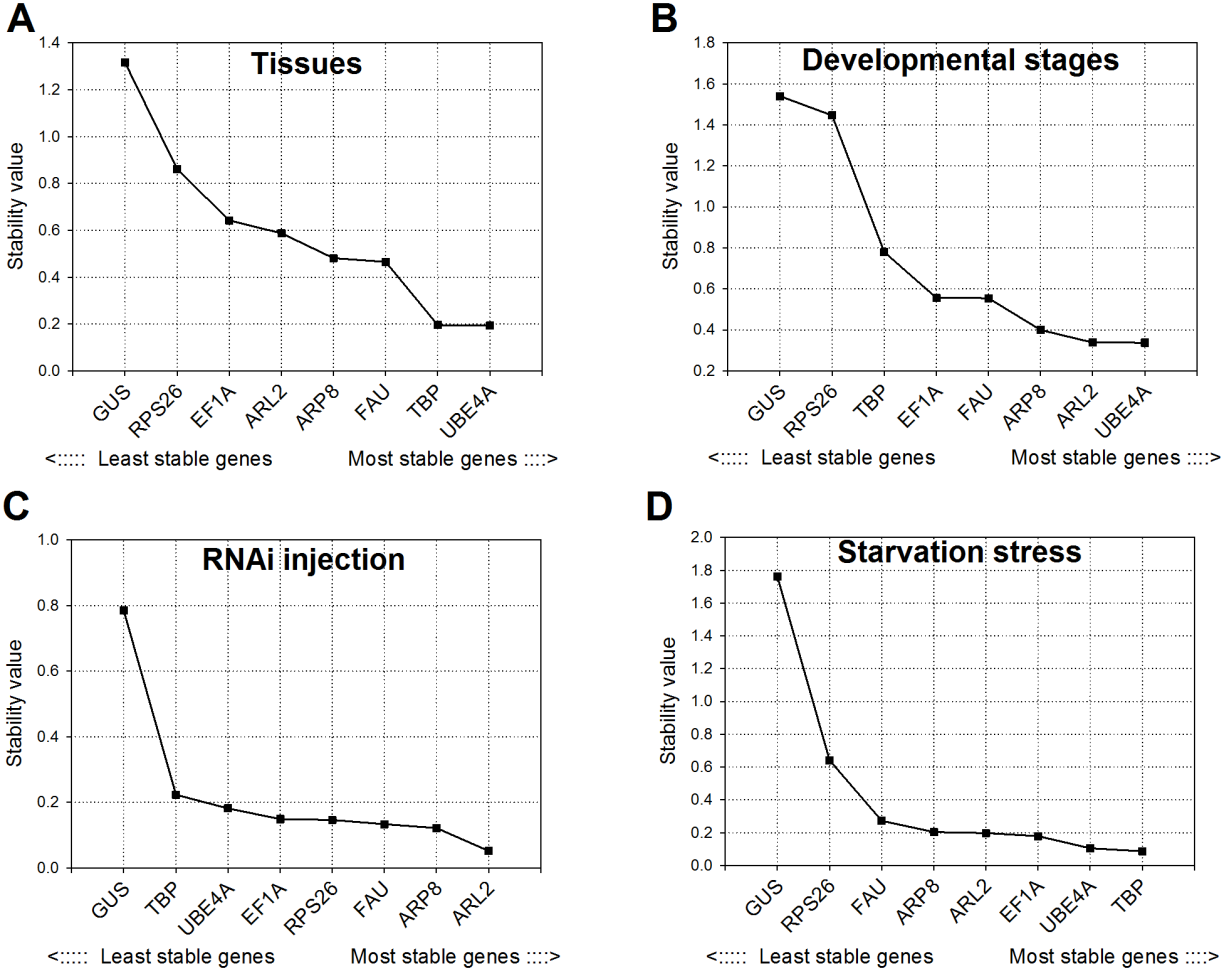
Pairwise variation (V) analysis of the candidate reference genes in brown marmorated stink bug using geNorm. The pairwise variation (V_n/n+1_) between the normalization factors NF_n_ and NF_n+1_ (shown along x-axis) is calculated to determine the optimal number of reference genes for normalization in brown marmorated stink bug under different experimental conditions as indicated. Each bar indicates change in normalization when adding reference genes stepwise according to rankings in fig 2.

NormFinder identified *UBEA4* as the most stable HKG among tissues (stability value 0.193) and developmental stages (stability value 0.337) (Fig 3A and 3B). The difference between *UBEA4* and next best HKG genes was small, however; *TBP* had a stability value of 0.195 in tissues and *ARL2* had a stability value of 0.338 in developmental stages. *ARL2* was also the most stable gene in RNAi injections (M value 0. 052) (Fig 2C). With the exception of *GUS*, other HKG genes were comparable to *ARL2* in RNAi injections. During starvation stress in *H*. *halys*, *TBP* and *UBEA4* were the most stable genes with stability values of 0.087 and 0.105 respectively (Fig 3D). Similar to geNorm results, the NormFinder also showed *GUS* as the least stable among tested genes in all treatments (Fig 3A–3D).

### BestKeeper analysis

BestKeeper showed that *EF1A* and *FAU* had the highest stability in tissues whereas *FAU* and *UBEA4* were the most stable in developmental stages (Table 3). For RNAi injection the most stable genes were *ARL2* and *ARP8*. During starvation stress, the *EF1A* and *TBP* were the most stable genes with standard deviations of 0.37 and 0.38, respectively (Table 3). Similar to geNorm and NormFinder analysis, *GUS* was ranked at the bottom with least stability (having the highest SD) among all tested genes across all treatments.

**Table 3:** BestKeeper ranking of eight candidate reference genes for for qRT-PCR studies in brown marmorated stink bug.

Rank	Tissues		Developmental stages		RNAi injection		Starvation stress	
	Gene	SD*	Gene	SD	Gene	SD	Gene	SD
1	*EF1A*	1.35	*FAU*	0.72	*ARL2*	0.17	*EF1A*	0.37
2	*FAU*	1.38	*UBE4A*	0.88	*ARP8*	0.18	*TBP*	0.38
3	*TBP*	1.57	*EF1A*	1.03	*TBP*	0.19	*FAU*	0.41
4	*RPS26*	1.96	*ARP8*	1.10	*UBE4A*	0.25	*RPS26*	0.41
5	*ARL2*	1.98	*ARL2*	1.26	*FAU*	0.26	*UBE4A*	0.47
6	*UBE4A*	1.99	*TBP*	1.34	*RPS26*	0.30	*ARP8*	0.52
7	*ARP8*	2.10	*RPS26*	1.63	*EF1A*	0.31	*ARL2*	0.56
8	*GUS*	2.45	*GUS*	1.98	*GUS*	0.89	*GUS*	2.18

*SD refers to the standard deviation

### RefFinder analysis

RefFinder compiles all the data from the three algorithms above to calculate final rankings. *UBE4A* was the most stable gene across tissues followed by *TBP* and *ARP8*. Both *UBE4A* and *TBP* were also the most stable during starvation stress. In addition, *UBE4A* was among the most stable genes across developmental stages, being only slightly outranked by *ARP8*. During RNAi injection in *H*. *halys*, *ARL2* expression was found to be most stable followed by that of *FAU* and *ARP8*.

### Optimal number of reference genes for normalization

**Fig 4:**
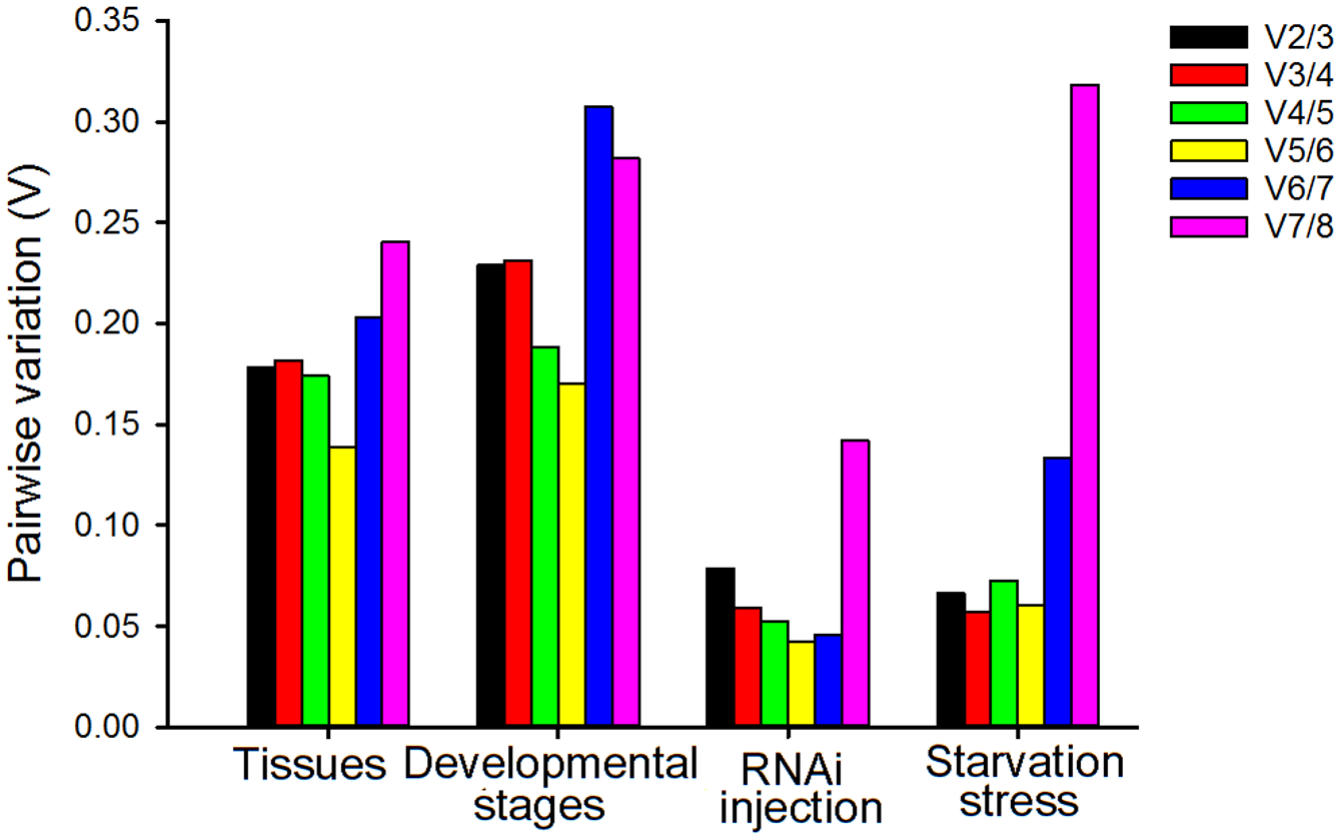
Stability values of candidate reference genes in brown marmorated stink bug as calculated by NormFinder. Stability values are indicated for brown marmorated stink bug under different experimental conditions as indicated in each Fig

Though a single and stable reference gene with moderate to high expression is sufficient for quantifying mRNA transcript levels, using more than one reference gene for normalization of gene expression data is suggested [21]. The optimal number of reference genes required for normalization under a given experimental condition can be obtained from the pairwise variation (V). Vandesompele et al. [21] proposed a cutoff value of 0.15 for V, below which including other reference genes is not required. For both RNAi injection and starvation stress treatments, geNorm analysis revealed a V_2/3_ value less than 0.15 (Fig 4), indicating that including a third reference gene will not improve the statistical significance. In various tissues, the pairwise variation did not reach the 0.15 threshold until V_5/6_. Across developmental stages, the threshold was never reached, although the V_5/6_ value of 0.17 obtained for the *EF1A*-*TBP* pair was close to the proposed 0.15 cut-off. Therefore, gene expression measurements across tissues and developmental stages should include at least three reference genes [21].

### Reference gene utility and target gene silencing in *H*. *halys*

**Fig 5:**
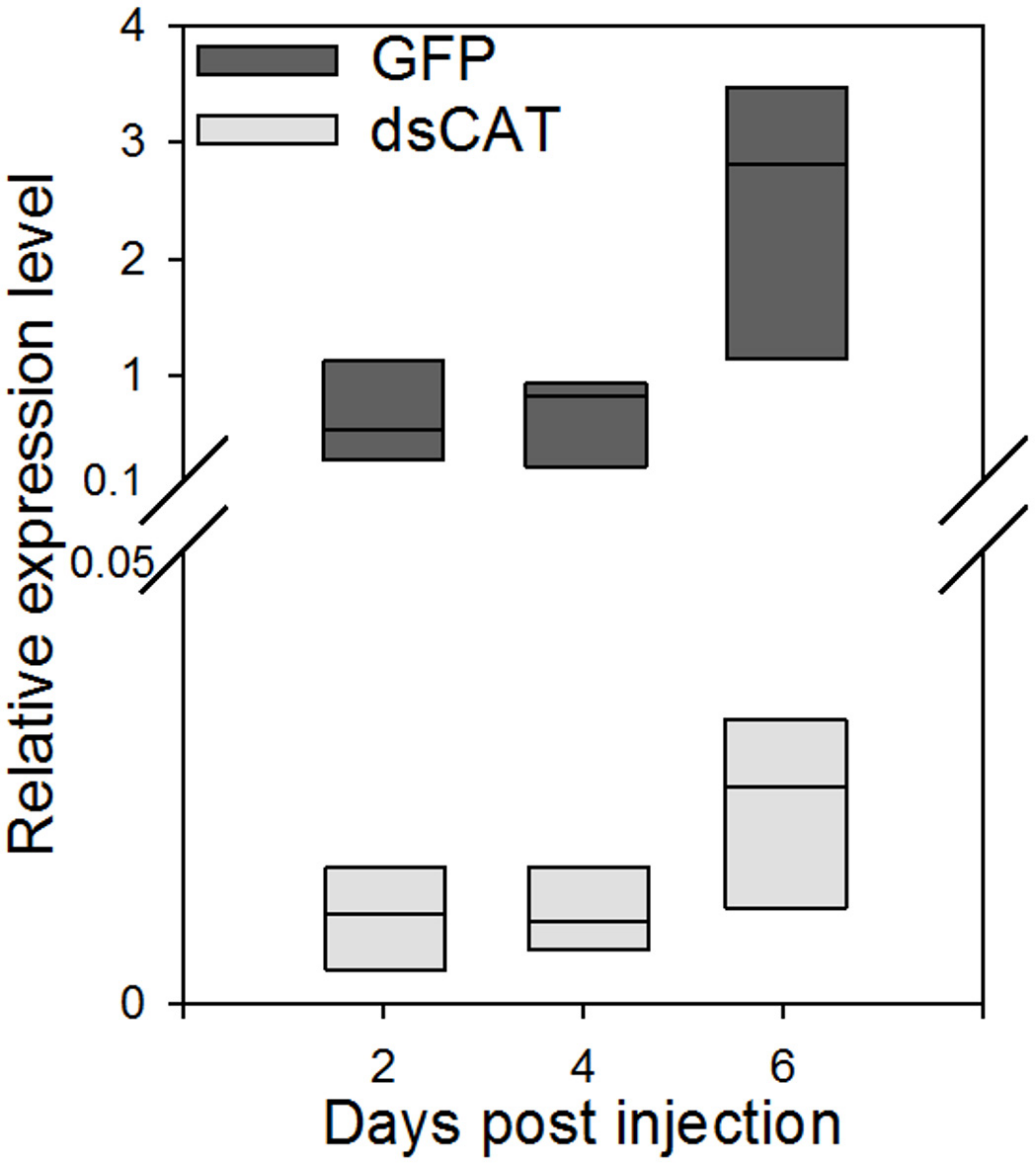
Effects of RNAi induced gene silencing on *HhCAT* expression in brown marmorated stink bug. Boxplots showing the distribution of *HhCAT* expression values measured through the qRT-PCR analysis in insects injected with *HhCAT* dsRNA (dsCAT) in comparison with those injected with *GFP* dsRNA are shown. *ARL2* and *FAU*, the two most stable genes during RNAi injections were utilized for qRT-PCR data normalization. Following the dsRNA injections, the *HhCAT* expression levels were significantly different (*P* < 0.05; t-test) compared to control at all three time points.

The expression of *HhCAT* was measured after injecting dsRNA. *ARL2* and *FAU*, the two most stable genes during RNAi injections, served as reference genes for qRT-PCR data normalization (Table 4). The gene expression data revealed a significant reduction (*p*<0.05, n = 3) in *HhCAT* expression compared to the control at all three time points following the injections (Fig 5). dsRNA injection reduced *HhCAT* transcript levels by 98% at all time points compared to the control. However, reduction in *HhCAT* expression did not result in significant mortality compared to control. Out of 30 total individuals, 13 died in the dsGFP compared to 18 for dsCAT injected *H*. *halys*. Average mortality (±standard error) was 60.0±0.33% after silencing *HhCAT* compared to 43.3±0.19% in the GFP control (*P* = 0.46). Despite higher mortality in the *HhCAT* silenced group, including 5 individuals dying on day 10, the Kaplan-Meier curves were also not significant (z = 0.89, *P* = 0.37, data not shown).

**Table 4:** RefFinder ranking of eight candidate reference genes for for qRT-PCR studies in brown marmorated stink bug.

Rank	Tissues		Developmental stages		RNAi injection		Starvation stress	
	Gene	GM*	Gene	GM	Gene	GM	Gene	GM
1	*UBE4A*	2.06	*ARP8*	1.86	*ARL2*	1.32	*TBP*	1.68
2	*TBP*	2.63	*UBE4A*	2.11	*FAU*	2.59	*UBE4A*	2.78
3	*ARP8*	3.03	*EF1A*	2.78	*ARP8*	2.63	*EF1A*	3.08
4	*FAU*	3.31	*FAU*	3.31	*EF1A*	3.64	*ARP8*	3.08
5	*ARL2*	3.34	*ARL2*	3.31	*UBE4A*	4.68	*ARL2*	3.25
6	*EF1A*	3.83	*TBP*	5.05	*RPS26*	4.90	*FAU*	4.82
7	*RPS26*	6.09	*RPS26*	7.00	*TBP*	5.66	*RPS26*	6.09
8	*GUS*	8.00	*GUS*	8.00	*GUS*	8.00	*GUS*	8.00

*GM refers to the geometric mean

## Discussion

Among the existing technologies for analyzing gene expression, qRT-PCR has emerged as powerful tool due to its high sensitivity, accuracy, specificity and reproducibility. The pre- and post-processing of mRNA samples for qRT-PCR analysis introduces variation which needs to be normalized using appropriate reference genes. However, qRT-PCR studies to date reveal a great variance of reference gene expression across samples (spatiotemporal and experimental treatments) implying that a universal reference gene cannot be used for all species or all experimental conditions [25]. Therefore, validation of reference gene(s) is mandatory prior to gene expression studies wherein a candidate reference gene should have amplification efficiency similar to target genes with moderate and stable expression irrespective of biotic and abiotic factors [26].

Ten reference genes that were chosen in the current study were analyzed by four statistical models (geNorm, BestKeeper, NormFinder and RefFinder) for a better evaluation of candidate reference genes by avoiding the selection of co-regulated transcripts [27]. The analysis of reference gene data sets in *H*. *halys* across biotic and abiotic factors indicated variation among the candidate reference genes as observed in several other insect studies [28–30]. Though *ARP8* expression varied among all the analysis tested, it showed moderate and stable expression across all the factors tested (Table 4). *ARP8* is involved in actin proteins—a major component of the protein scaffold that supports the cellular cytoskeleton—and is moderately expressed in other insect studies [26,31–33]. On the other hand, our data revealed *GUS* as the least stable, which is in agreement with other insect studies [34]. Also, it is surprising to note that *RPS26* performed poorly in the current study as RPS family genes (putatively involved in protein synthesis) are well documented for their utility in insect gene expression studies [32,35,36]. Overall stability patterns of various genes clearly indicate that the expression stability of housekeeping genes were affected across the biotic and abiotic factors (Table 4).

Following the dsRNA injections, all genes except *GUS* showed relatively a stable expression though with minor variations (Figs 2 and 3, Tables 3 and 4). However, we don’t rule out the changes in the relative stability of these reference genes following the treatment with dsRNAs of different target genes. As such, we observed a robust RNAi response in *H*. *halys* (Fig 5), with greater than 98% reduction in *HhCAT* expression. Interestingly, the silencing of *HhCAT* did not result in significant mortality which is in contrast to results seen in other species where *catalase* silencing has resulted in significant mortality [37–39]. *Catalase* is an important gene which responds to oxidative stress, and, indeed, *H*. *halys* increases *catalase* expression upon immune stimulation (*i*.*e*. tissue puncture, which would be similar to dsRNA injection) [18]. We ended our observations at 11 days, with 5 *HhCAT*-silenced individuals dying at 10 days. It is possible that, given additional time, more *HhCAT*-silenced individuals would have died, consistent with the importance of this gene seen in other insects.

Overall, the current study has identified stable reference genes across various tissues, developmental stages and treatments, including starvation versus fed and during dsRNA injection in *H*. *halys*. Taken together, using different software algorithms and considering comprehensive analysis results, we recommend that the following gene pairs are the best for use as reference genes under specific treatment conditions in *H*. *halys*: 1) *ARP8* and *UBE4A* are the most suitable reference genes for gene expression studies among tissues and developmental stages; 2) *ARL2* and *FAU* should be used for dsRNA treatments; and 3) *TBP* and *UBE4A* as reference genes for starvation treatments. In addition, this study reveals a successful gene silencing through RNAi in *H*. *halys*.

## Supporting Information

S1 Fig Reverse transcription PCR to test primer specificity. Results of RT-PCR (35 amplification cycles) are presented for primer pairs used to amplify candidate reference genes in brown marmorated stink bug. Details on primers and product size are provided in Tables 1 and 2.
(TIF)

S2 Fig Melting curve analyses to test primer specificity. The melting curves are presented for primer pairs used to amplify candidate reference genes in brown marmorated stink bug. Detailed gene names are provided in Table 1.
(TIF)

S1 Appendix The transcript sequences for genes described in this study. (PDF)
