# Supplementary figures and images for: Quantitative RT-PCR Gene Evaluation and RNA Interference in the Brown Marmorated Stink Bug
Source: PLoS One. 2016 May 4;11(5):e0152730. doi: 10.1371/journal.pone.0152730 (PMC4856283; doi:10.1371/journal.pone.0152730)

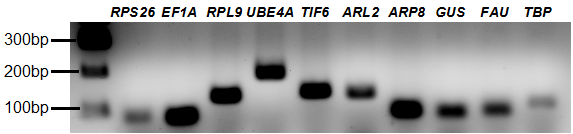

Supplement: S1 Fig — Results of RT-PCR (35 amplification cycles) are presented for primer pairs used to amplify candidate reference genes in brown marmorated stink bug. Details on primers and product size are provided in Tables 1 and 2. (TIF) [file pone.0152730.s001.tif]

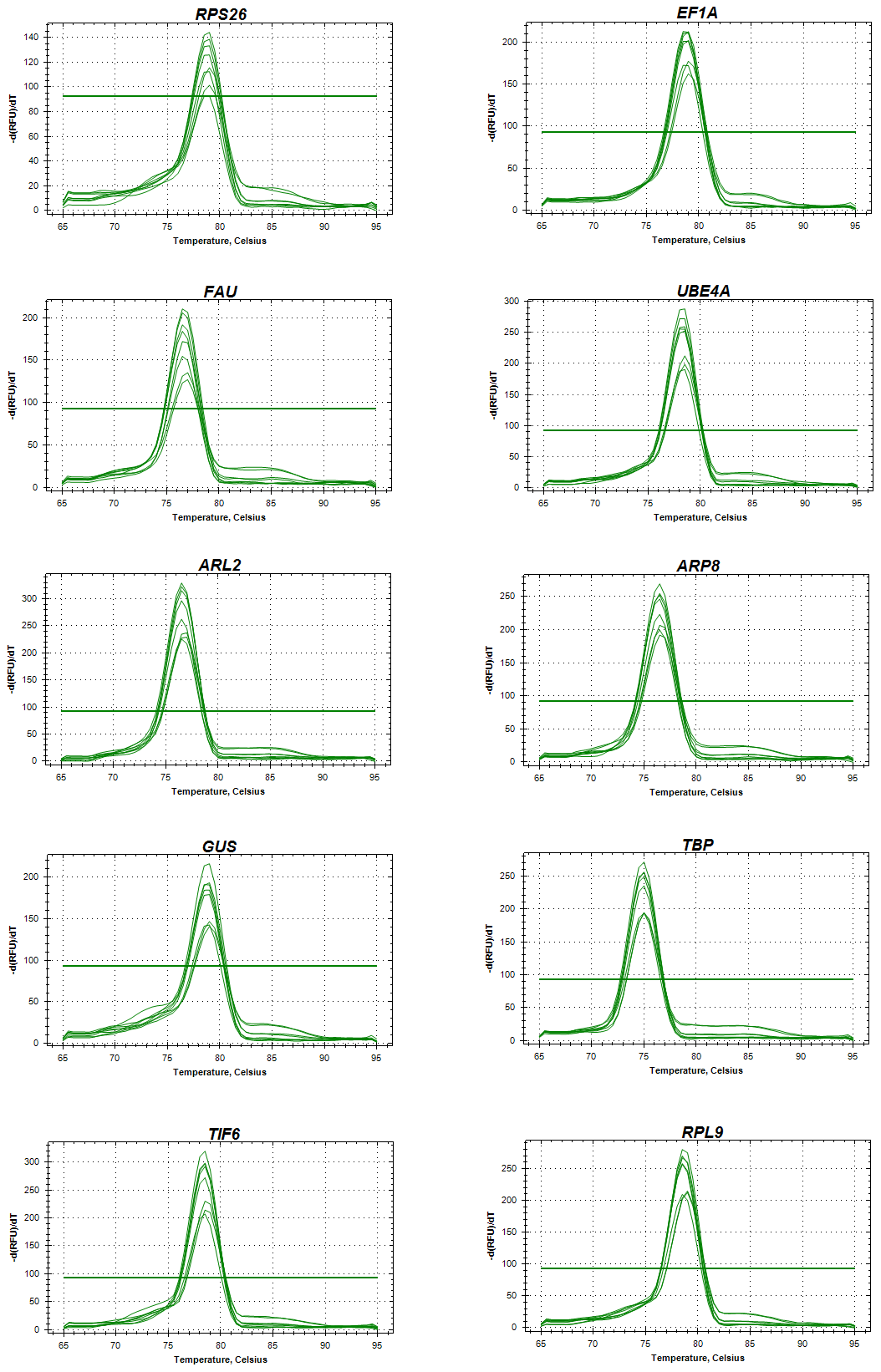

Supplement: S2 Fig — The melting curves are presented for primer pairs used to amplify candidate reference genes in brown marmorated stink bug. Detailed gene names are provided in Table 1. (TIF) [file pone.0152730.s002.tif]
