# Supplementary material for: Quantitative RT-PCR Gene Evaluation and RNA Interference in the Brown Marmorated Stink Bug
Source: PLoS One. 2016 May 4;11(5):e0152730. doi: 10.1371/journal.pone.0152730 (PMC4856283; doi:10.1371/journal.pone.0152730)
Supplement: S1 Appendix — (PDF) [file pone.0152730.s003.pdf]

>BMSB_*RPS26*
GTTTTTATTAACATTAGTTTGAAAAAGATTATTTTCTTATCTGGGGTCCACCTTGACGCA
TCATATCCCTTGGGAAGTTACGCTGAGGAGGTGTCCTAATTCTTCTCTCAGCCTTAGAAC
GATTACGTACTACTTTAGAGTGGATCGCACATGATACACAATAATGCAGTTTAGCGTAGA
GTTTTGGCAATTGGTATGATGAGTAAACACTAGCATCTGTGATGTCTCTCACAGCTGCAG
CTTCTACAATATTACGGATAACGAATTTCTTGATAGCCTTATCCTTTGGTACACAACGGG
CACAGTTGGTACAACGGACTGCTTGTACATGCCCCCTACCGTGTTTAGATCTTCCTCCAT
TCCTCCTTTTCTTGGTCATTGTAAAATTATAACAGATATACCCCGAACACGAAGCACTTG
CTAAAGAACCCTACCAAAGCCTTCTGAATGAAAGCTGCGCACTGTGTCGCTCTCTAGCTT
ACAAACTCTTATGGCAATTACGGTATATTTGTTTTTATCTATAATAATAATAATAGATAA
TATCTGTTTAAATTTTA
>BMSB_*EF1A*
GGGCTAGAAAAATACAAATATATATATTCAAATAAATATAAGAGAAGAACTATTCCTCGA
GGGACTACTTTTACCCCCACTACTTGCCAACGGGGCCACTGGTAGGAGTTGTGCGCATGT
TGCAGTCGTCTGTCTTCCTCTTTCTGTCCTGGCATTGGGAGATATCTTAACGGTCATTGT
TATATAAACTGCTAAAAAATGCCCAAAGAAAAGATTCATATTAACATTGTTGTCATTGGA
CACGTAGATTCAGGTAAATCTACTACTACTGGCCATCTCATTTACAAATGTGGTGGTATC
GACAAACGTACTATCGAAAAATTCGAGAAAGAGGCTCAGGAGATGGGCAAAGGATCTTTC
AAATATGCCTGGGTATTAGACAAGCTTAAAGCTGAACGTGAACGTGGTATCACAATTGAT
ATTGCCTTATGGAAATTTGAGACTTCCAAATACTACGTCACAATTATTGATGCCCCTGGT
CACAGAGATTTCATCAAAAACATGATTACAGGAACTTCTCAGGCTGATTGTGCTGTGTTA
ATTGTAGCTGCGGGTACTGGTGAATTTGAAGCCGGTATCTCTAAGAATGGACAGACTCGT
GAGCATGCACTTCTTGCCTTCACACTTGGTGTAAAACAATTGATCGTAGGAGTTAACAAA
ATGGATTCAACTGAACCTCCTTATTCAGAGAGCAGATTCGAGGAAATCAAAAAGGAAGTA
TCATCCTACATTAAGAAGATTGGATACAATCCAGCCTCCGTAGCTTTTGTTCCTATTTCT
GGTTGGCATGGAGACAACATGCTTGAGCCATCTGACAAGATGCCATGGTTCAAGGGGTGG
GCTATTGAAAGGAAAGAAGGCAAAGCTGATGGTAAATGCCTTATTGAAGCTCTTGATGCC
ATTTTACCTCCTAGCAGGCCTACCGATAAGGCCCTCAGGCTTCCACTTCAGGATGTGTAC
AAGATTGGTGGTATTGGAACAGTGCCAGTTGGGCGAGTGGAGACTGGTGTCTTAAAACCA
GGTATGGTTGTCACTTTTGCTCCTGTCAATCTTACCACTGAAGTAAAGTCTGTTGAAATG
CACCACGAAGCCCTTCAGGAAGCTGTACCTGGTGACAATGTTGGTTTCAACGTTAAAAAC
GTCTCTGTTAAAGAATTGCGTCGAGGTTATGTTGCTGGTGACTCCAAAAACAATCCACCC
AAGGCTGCTTCTGACTTTACTGCTCAGGTTATCGTATTAAATCACCCTGGTCAGATTTCT
AATGGATACACTCCCGTACTTGATTGTCACACTGCCCATATTGCTTGCAAATTTGCAGAG
ATCAAGGAAAAGTGCGACCGTCGTACTGGTAAATCTACTGAAAGCAACCCCAAATCCATC
AAGTCTGGTGATGCAGCTATCATCAACTTGGTTCCAACCAAACCAATGTGCGTAGAGTCA
TTCCAAGAGTTCCCACCTTTGGGACGTTTCGCTGTGAGAGACATGAGGCAAACTGTTGCT
GTCGGCGTCATCAAGTCTGTAACCAACAAAGATATAACAACGGGTAAAGTGACAAAGGCC
GCCGAGAAGGCACAGAAGAAGAAATAACTAGGTGTCGTCGTCAGATCAGCAACAGGCAAG
CCAGAACAGCCACCGCCATCAGAATCAGGAAGCTATTACTTAAAACAATTATGTCGCTTT
TCTCCCCATCAGCAGCACCTATCCAAGTGGCTCCTGCAGCCGTGTTTCCATCGTAAGGAG
TTCCGGAGGAAAAGCGGCTACATGACCTTCACTTCTCTCAAACCTTTCCTTATATATGTT
ATTATATAATTACTTATATAATCTATATTATTATATACATAGTATAGCATTATAATTAAT
TCTTCTTAAATACAGTTTTTTAAAACTCACGTGGAAGGCTTTACGATTTGTTTTTTCTAT
TCTTTTTTATTTTTAGAAGAGGAACTCGCGTCATGTGAATAGCATGTTATTTTCATTTTA
TTTTGTTCGACAGATATTTAATTTATATTCGAAGATTTTTAAGTCATCTTTTTTGTTTTG
TGAAGCAACGGTAATTTCTGTATAGATGGTGATAACAATAAATGCTTCATGACATTGAAA
AAAAAAAAGATGACTTAAAAATCT
>BMSB_*RPL9*
TTTTTTTTTTTTTTTTCAAATTTTATTAAAACTCATTTATTCATCTTGTACAACAGTTGT
CTTCTCGGAGACATAAAGACCATCCAAAAACTTTCGGATATCCTTATTTTTGACAGTCGT
TGACTGTTGGATGAGGGCAGCTGATCTTGAAACATCTTCAATATTGTTACCCTCAATAAT
AAGTTCGTCCTTCTGTTTTGTAGAATTTGTAACAGTAACACCTGGTGACATTTTTACCTT
CCTAATATATTTCTCACCAAGAAAGTTACGGATTTCAATAACGGAATTGCTTTCCGATAT
GACGCAGTTGATGGGAAAGTGAGCGTAGACTGCTCTCATTTTATATAAGAATCCCTTTGT
AACACCTTTAATCATGTTTTCAACATGGGAACAGACAGTTCGAACTGCAGCAAGTTCCTT
TTTGGTGCCAAACCATTTTTCAACTTTAAGCAGTTTAGAATTCACCATATGGATATCTAG
TGCTAAATGCCTGAATGTCCTTTTCAAGACACCCCTAGGTCCTTTAACAGTAACAATCCT
GGACTTCACATGGCATGTAACCCCATCGGGGATTTTTACGGTTTGATTAACAACTATTTG
CTTCATCCTCAGAGAGGTTAGGCAACCGAAAAAGAACTCTAGCACGCCAACAGCAAAGAA
CTCAGCAAAGCCTCTTCGATATAGTACTACGAGTATAGTACACCTCCATCTTAAAAATTT
ACAAAGAGGTATGAAACTGAAACCACCTTCAACTTTAGATCGTACTTATTACAATAAATA
ACATATCTTTTAAAGATGCTGCTACACGTTTGCCAAA
>BMSB_*UBE4A*
ATAAATATTAATTTATTATATACTTGTAATGTACAACATGATACAGATAAATATTTATAA
TGTCATTGTTTATTCGAGGAACTTGCTTGCCTCTTCCTTTCTGCTATCCAAGTTTCAATC
TTTTCCCTAAGTTCGGTATTCGATTTGACCATATTCATTGTTAGAGGTGATCTGTTGAAT
GGGTCCGTTTGATCGCTCAGCAAGTGTCGTGCGATAGTAGACCTATCGATGTTAACCCTT
GATGAAGGAAGTATAACAGGGTCCGTCATTAGCGTTGACATAATCGGATCCAGGAATTCC
TCTGGTGCTTCGGACAGCAGTTCCTCATCACCTTGTTGAATAGATGCCAATCTGGCCACT
TCTTCGGACACTCGTTGCAGCTCCATCACCATTTCCATACCGCCACCGACACGAACCAAT

ACATCACTTGCTAATGTAAACAGTTGAGGGCTGTACGAGCGACCATCCTTAGAGACAGCT
GAGCAAAATTCAGTACTAGAACCTAAATGAATATAAATACCACAAATGTCTTTGACTATC
GCACCAGGGTTAAACATATACTCTTCATGATCTTTTACCTTCAAATTTTTCTTGTTGGGT
CCAACCAAATGATACAAAAAGTAATTGAGCATGGCAGTAATCCTTTCGACCATCATCGGA
TGGCAAAAAATTGACTTAATTTCTGAGCTCATATATTCTAAAGTGTGTATCGTTTCTTGG
CCTAGAATGTTATCAAACCTCGCTGTCATTCCTATTTGCCTAAATATATGCTCGTTACGA
TCTCTCTCGTCCAGTGGCAGCCTTTCCCACTCTCCTGCATCTCTGGCTGTTTGAAGAGTA
CGCAATTGTGCCATATTAGAAAGAGCTTCGTCGAGGAGAAAAATGGCATCGTTCATGAGG
AGGTTGAGAAACCTGAGGAAGAGTGGAGGAGTCACTTCTTCCATGTTTATTTCCGCATCT
CGAGCGAGCTGTTTAAAAACATCTCTGTGCTCTTCTATATTCCATAGATAGTCCATGACG
GTGTACATCGGCCTTCTGTAATTGAACTTCTGTTCGAAAGCGACGCCCTCTCCAGTCATT
TCAATTGAGACGAAAACATTTATAAGACAAACAACAATTTGGGTCTTGTGAGGGTGATCG
ATAAAAAGCCTTTCCCTGTAGAAGACACCCAGAGGATTCGGGTCCAGCGTAGGAACTTGC
CCTTGATCCCTCGGCAGGAGGCATTCGAGGGATTGAGCAAGCTCTGCGCGGAGGTGAGGA
TTGCTGGCTCGTCGTTTGGAACCCATCATGACCAGCAGCAGCGACAGCAGTGGCTCCATC
AGGGCGAGTCCGCCTTCCTCAAGTGATTGCGGAGAGTAGGACTTAACCAACTTCAAATAC
CATGCCACGCTTTCCACTACCAATTCGGGTATGCATTTGAGTATCGGGGGTCCATATTCT
GGCAGAGGAAAAGTCAGCTGGCGGTGAACTATTGGTGTATAACAGTTCCTTTCCTCGGCG
ACAGGGTCAAGAATTACTTGTATCAGCCAAACAGACGTAGCCACTTCAAACTGTCCCAGC
ATCCTCACCATCATTGGTTCAAGAAGGGCACATCTCATACACAAGTACCTGGACATCTCC
ATTTCCATCCTTTCTCTGAATGTTTCTACAATTTCCTCTCTCGGTTGAGATCTGGCCTCA
TTTAAGGCCCTCTGTAATCTTCCGATTTCCTGATAAAGTTGCTGAGTTCTTTCGAGTAAA
ACCCGAACGCTGAGGTCTAATGCTCTATGAGTAAGAAAGAAACATTCTGTTATAAAAGAG
AATGAAACAGTTGAAAAAGGTTTCTTCTCTCCAGACTCAGTTGGTTGTAAACACGTTTCA
TTATGCAACGTCGTACAGTGAACTCCTGCATTATCCATTGCTTCTTTATTTGTTCGGTCC
ACCTTAAAAGCGGGATAAGTAGGATCGATTTTAAGAAGTTTCCTTTGGTCTTTAACAAAA
GGCTGTGAAAGCCTTAATAGTACTGATGCCAAATTTATTGAAAAGCCATCACTGACACAT
CCGACCGCTCCAGGGAGGCCTAAACTTGTAGTGGCTGAGATGCGACCCCTGGGAAGATTT
GAATCCAAGCAACCTCCTATCCACTGAAGAACTTTCTTCCTAAGATTTGGTGAACCTCGA
AGAATCTGTTCAATAAGACTATGGAACCTTTCACATAGTTCTCCCATGGCTGTCCATAGA
TTGTTGTCTAAAGCGTTCAGAGCAGCTTCCAAGGGACTTTCAAAGTATTCGATAAATCCT
TCTACGGTCTTTGGAAGACAAGAGAGATTAAGTATCACTCCAAAAAGCGTCTCAGCATAA
GCTAAACCCCTTTCGGTATTAGCAGGCATAGAATATGTAACAACGACCTCTGCTAGGGCT
GGGATGGAAACAAATGAACTTAATAAAATAAAATGAGATCGAGGAAAATTTATAACTAAG
GCACGAGAAAAATCTAAATGAACTTCATTTAAAATAGGGCTAAATGCATCAAACAAAGTT
CCTTCATCTTCGTTTAACATTAATTGAACCAAATCATTTACAAAACTGGTAACCTCGGTT
GTATCTTCTAGTTCATAAAGTGTAATGACCTGTCTATAAATATTTTGCATTTCAAATAAA
GCAGGCTGTCTAAGAGCAGTAGAAACATTTTCCACCACGTAAGCGGTTAAATTTTCCAAA
GTTTCTTTTGGAAGATTGTTCGTTTTACTTCTTATCAAATTGTTCAATCGTTTGAAACAT
TCAAATAAATATAATATACTATGAGTTTGAATGACGTGAGCTTCGGTTCTCTCATTTCTC
AGTTTCACAACTTTGCTTTTAATTAAATGTTTTGAAGGATCAGTGAGTAGAATCCTTTCA
AAAACAGAAAACTTCAGAGTGTCAATGTCAAATGTTGCCGGAGTTCCTTCAATATATATT
AACTGTCCTATTCTACCTGAGCTTAAAGATAAATTTTCTTCCTGAAGGGTGATTCCAAAA
ACATCTTCAGAAATTTTATTGATCTCTTCAGAAATTTGGTCTTTTTCCGCAACCCCACTC
TCAGGAGGTTTATCGGATTTAGTACTGTTTTCCCCATCAGTATTACCAAACAATTGGTAA
AAGGGATTATTTCCATTATCTTCAGACATCTCTTTTTTTTTAAGAAAATATGATTTTAGC
TACATATATACTTGTAGAGGTTAGGTTTTAGATAAAAATAAAAATATAAATAATAATACA
CCACTTCCTGAGTCATTGAATATTGCACAACACAATGATAATCACTCACTCATTAATTAA
TAAAATCAGAAGACGACCTAATTGCTGTCAAGCACTGTAAAACAACTTGTGAAGGTTGTA
TTCAGAATTTACATGTAGTACGATCCATTATATGTTTCATGTCAGATATGAAATAACAAT
AAGAGGATTTGTAGTCTTGAGAGCTTATTAAACGATTGAAACTAGGCATATCACAGTTTA
TCGAAAAAGGAATGTGTCCCTTAAAATGATATTCATGATTTAAACGTATACTAACACTAC
ATTGGCGCCTGAGGCGCTAATGGTCTAGAAACAGATTTAAGATATTGTTGATAAGGTAGG
TTATCAGATTTTTCATTATCACTTCTCAGTTACAGCCCGTACTTGACAATTTCGGACATA
TT
>BMSB_*TIF6*
TAGCATGATACTTTAATCAACCTTCCGTTTCCCCCATCACATAAATGAATTTGACACGCC
TTCAAGTGCATATTACAAAATTATACTTCATAGCATGATACATACTTCTGTTTAGTAAAA
CATTTATTTATCACCAAGGAATGTTTCTCTAAAGTTGATTTCATTTAAGCCATACTGTCG
ATCAAAGAAGCACGCATTGTTGTAGTAATTGTAGCAGGCTGAGCTTCATTTAATTTGAAA
ACACTTTCAATTACAGAAATTTCAGTGGATGTAGTGTCCAAACCACAAAATGCTGACCAA
TCATTCACTACCATACCAGCGGCAATAACTTCTGATCCCCTATTTATTGTACCTGCGACA
AGTGGAACTTGTAGTAATGAGGATAGTTCATCTTGGTCTTGAGAAGAAGTTTGTGGATGA
ACCAGACCACCTTGATTTGACAAAACACAGTATGATCCAACTAACAGGTTTGAAGCTACG
GTTTGTCGAAATACTTCGACATTCAGTGTATCGGACAAAATTTCTTCTGTTTCTCTGTCA
AGATCAGGATGTACAAGGGCTACATAATCATTACACGTAATTACATTTCCTAAAGCTGAT
AATCTCTCTTCTACCCTTTGTAACTTTACAGATTCAGGTAGAGAATTCCTTAAATGTTGA
AGCTCTGTATCAGTAGTTGTATTAGGAACAAGAAGCCCATGTCTATTACCAACACACATC
CTTCCAATTATTCTGCACCCTGCTAGAGAAGCATGAATAACGGGAATAGTTTCTCCTAAC
TCTGCTTCAAAAACACTGTAAAAATTTTCTGAACCACCTATTGCAACTAAACAGTAAGCA
TTAGTTAATTTACTAAAAACTCCGACTTCATTATTATTTTCGAATTGCACTCTAACAGCC

ATTTTAATAATAAATCACAACGAATTTGGTTAAGACACTTAAAACCATGTATTCAAAAAT
CGACACAATACCATGCAACAAAATATAAACACGTGCGGCAACACGGCATCCAATGGAGTT
CTTAAAGATTA
>BMSB_ARL2
CGTCCACGTAATCGTTGCTCTAAGTATATTCTGCCACCGAATTATTTCACTGTGTACTTA
GTTCTCGTGACTAATCGTCGTGGAGTCATTTTAAAAATTCATATCTTTATTTTTCAATTT
TGTCTGTGTTTATGATATATTTTCGGATCACTGAATCTATTGTGCGCTACTGGACAGGTA
TTCAAGTGTTACTTTTGAAAAGATTAGTGTTTCTTCAACCATCTTAATTATGTCTTTCAG
TTTAAGTTATATTATATAATAAGTTATGCTGATTGAAGATATTGTCTGTTAACTAACACT
AATTTTCAAACTTTTGTTTGTGGTATTTATTTTTCACCAAAATGGGCTTAACTTTGTCAA
GTGTTTTGGCACGTCTCTATGGGAAAAAACAGATGAGAATTCTTATGGTTGGATTGGATG
CTGCTGGAAAAACAACTATTTTATACAAATTTAAGTTAGGTGAAATTGTTACTACAATTC
CTACCATAGGTTTCAATGTTGAAACTGTTGAATATAAAAATATTTGCTTCACAGTTTGGG
ATGTTGGTGGTCAAGATAAAATAAGACCATTATGGAGACATTACTTTCAAAACACACAAG
GATTAATATTTGTTGTTGATTCTAATGATCGAGAAAGAATTCAAGAAGCAGAAAGAGAAC
TTCAAAACATGTTGCAAGAAGATGAACTTCGAGATGCAGTTTTGCTTGTTTATGCTAATA
AACAAGATCTTCCTAATGCTATGAACCCATCAGAACTTACAGAAAAGTTAGGTTTGAAAC
AGCTTAGAAATCGCCAATGGTTCATTCAGGCTTCATGTGCTACCCAAGGACATGGTCTAA
CCGAAGGCTTAGATTGGCTTTCGAATGAGTTAGCAAAGAGATCATAAGAGCCAAATAAAA
GAAAGAAGTACTGATCTTAAGAGAAGAATTTGATGTTGAGCATTTTGAATTTCATCTATG
TAAAATTAAAAGTGTTTAGTGACAAGGTTAGTCTAAGAACATTGTTAAAAGAAGGATTGA
CATTCCAACTATTAATGTCCTAAGCCTAAGCATATGATTAAGAAATACATACAAAGTGTA
AGATTTGGTACCGATATTTTGGTAAAAAAATAAACAAAAAACAAACCATATTTTCAACAA
AACTGATATACCAAATTCTCTATACAAATTTTGTTATTTTACAGTTTACTTATGTATTTG
CCTTTGGCTTTGTATTATGCATGAATAAGTATATTGTTTCCTCTTTTGTAACTGATTGTT
ATTGGTTAGCTAATCAATGTGTTTGCAGAGAGAGATCATGATTTTATAATTAATAAACTT
CATTTTTTCTTTGGATGTATTTTATTATTAAAGAAGTGACTGATAATAACA
>BMSB_*ARP8*
CTTTCAACGTGCCCACTTGTGAAGTCGTTTCGGGATAACCATTTAGCACCTCTATTGGTT
ATAAACCGAGAAGGTTTAGTTTACAAATTTTAGGTCTTTAATTGTTAATTTTTTGTTTAA
AATGACTACGATTATAGAACACATTGAAGAACCAGTGCAAATGCAGACGCAAAATATAAT
CATCATTCATCCAGGATCCCTTTATGTCAAAATCGGTCGTGCATCTGACTTGAATCCTTG
CACAGAACTCCATGCAGTTGCAAGAAAAAGGCTTCCTGGAGGTCTTGCTCACCAAGATCC
AATATTGCCTCCTCTGGTTCCCAAAGTGAAGGATCTTGAAGCTGAGATGGATGCTTGTCG
GCTTCAGGTGTCTCATACCCTTGGTTCTTGTACAATGTCCAACAATCAGCGAAGGTATGT
TACCCCACCTCAGCAAATATCAGCCTTTAATAAAAGAGTTATTCCTGAAGTAGTTGCTGA
AATTGGTCCTAGCTGGGTTAAATCAGAGGAAAACGTTGTGTTTGGTGATGGTGTTTTGCA
TCTTAATCCTGCTTTGGGTTTTAATGTTCACTTCCCTATGAAACGAGGTGATCTTAATAG
ACATTCAGGTGTAGGAGGATCTCTGACTGGTGTTCTTGCAGATTTGCAAGCTATATGGTC
ATGGGTCATTAGTGTTCGATTGGGTATACCTCTTCAAGATTTAAAGAATTATAGAGCTGT
TCTTGTTATACCTGACATTTATAATCGACAATATTTAAAAGAATACGTGACACTTTTACT
GGATATGGGATTTTCCTCATGTTTCATGCTACAGGATCATGTAGCAGCCACATTTGGTTG
TGGGGTTGGGACGGCTTGTGTTGTTGACTGTGGACATTCTAAGACATCAGTGTCTTGTGT
TGAAGATGGTATATCTCATCCTGCAACTAGAGTGCGCTTACCATATGGTGGAGGTGACAT
AACACAGTGCTTTCACTGGCTGCTTAAAAAATGTGGATTCCCTTATCAGGCAGATCCTGA
TTCATTTCATGATAATATGTTGCTTGATAAACTGAAACAAGATGCTTGCCATATTGACTT
AGATATTTGTGGTTGCAAGGAGTTTACATTTACGGTTAATAAACCGGAATCACCTGTTTA
TAAATATACTATTCAGAGTGGTGATGAAGCGATCATTGCTCCATTATCTTTATTTTATCC
AGAATTGTTGATGCTGACTGGCCCTAAGCAGATATCAACTCAAGGGGCTAATACAGGTGA
TTCTACTGATCCACATGATGCAGATTATTTACGAGAAACAGGGAGGAAGAAGGAATGTGA
AGTTGAAGCAGACGAAGAGCTCGCTGAAGTGGAGGCATCTGGCTCAGGAGAGTTCTGTAC
CAACGAAGGAGGCCTTCTCCCTCTTGACCAAGCTATTTTACAGAGCATACAAAAATGTGG
CTCTGAAGATCTAAAAAGAAAAATGTATTCATCTATACTTCTCATTGGAGGAGGTTTGAA
ATTCAAAGGAATGATATCATGGTTAGAGAAGAAGCTTTCTCAACAAATTCCTATTCCTCC
AAAAGATGACGGTGAGCGAAGTGTAATGGAGGGAAAGGATGCTGCGGCTGTTTGGAAAGG
GGCTGCCATTCTTGCCTGTCTCGAATCTGCTGGCGAACTATGGATTTCAAAAGATGAATG
GGTTAGGTTTGGAATAAAACTGATACGAGAGAGAGGCCCATTCCTTTGGTAGCTAATAGT
CTTACCTTTAATTTGTATATTATC
>BMSB_*FAU*
CTCAACTTGAAAGGAAACATTCTAACTAAGAACATAAATGTGTTCACTACATGTTCCATG
GAGTGATCACAATGGCATAAGCTCTACGAGCCAAGTCTCAATCAAACCTACTGGAGAACG
GCCATTTGCTTAGAGAATTTGTTGGATCAAAATGATTCTCGGAGAGGAAAAGATCAGCCT
TTTCGTTCGTGGGCAATCAAACCATTTGGTAGAATGTTCACAATCTGACACACTAGGATC
TATTAAGGTCCGAGTTGCCAGCATTGAAGGGATACCTTATGAACTTGTTAGTTTAGCAGC
ATCAGGAACTCCACTCCAAGATGATATGTTTGTATCTGATTTGGAAAACAGAAATCTTGA
CCTCACAGTTCCTTTACTTGGAGGTAAAGTCCACGGTTCACTGGCTAGAGCTGGTAAAGT
GAAGGGTCAAACTCCAAAGGTTGAAAAAATGGAGAAGAAAAAGAAGAAGACTGGACGTGC
TAAAAGGCGTATCCAATACAACAGACGCTTTGTCAACGTTGTACAGACTTACGGTAGAAG
GAGGGGGCCTAACTCTAGGTCTTAAAACA
>BMSB_*TBP*
TTTCTCTTTCAGCTGGTGGACCAGCATACTGTAGTGCTGAGAGGTATTTACGAGATGTGA

ATTTCTCTACTTCAAAGACCCAGTGACCAGTCAGTGTTGGAGGGCAAACCACAAGAGAAG
GTAATGGTGCACAATCAGCCTTCTTTGTGTCCTTATACTTTAGTCTCCTATCGTAATGGT
CTCCAGCAAGAATACAGATAGATTGGAGAGTCTTTCCAAGCCCCATATCATCACAAAGTA
AACCGTGGAGTTTATACTTATTAAGAAAAGCTAACCAATTAACTCCAGCCTGCTGATAGG
ACCGCAATTCAGCTGAAATCGGAACTGGAACTTTGTAATCGGTTATAGTAGATGGGTTGA
AGAGCTGCTCAACAAACTGGCGCTGCTCATTTTTTAATACCAACAGCCTAGGGGTAAGTT
TAGGTGGGTTGGGAATTCCACTTTCTAGAGGCATAACCTGAATCAAATCTGCAAATGTAT
GAGTAGCAAGAGTTCTGATGGTTTCATTTGGATCGCTCATTCGTCCTAATAGTGGTACAA
TGAGAAAAAATACATAAGGCACTACAACCAATTGTAATTTTTCAATAATAACAGTAACTG
CTTCAATAGAGCCTCTTCTCTTAGTGTCATCGTTAACAGCAGACAACATAGGTATTACAA
AGTCAATCACTACATCCATCACTGTATTAGGCTCTATACTTGCAAGTTGTGCTAAACAAC
GTGAAGCCATATGTCTAATAGCAGAAAAGGGATGGGAGAGTAAACAAACCAAATGGTTTA
AACATAGAAAAATTTGGGGTAAAAGAGATTTATCAACTGAAGGGCAAGTAACTTCTAAAA
CCTGTAGAATCCCGATTAAATGTTCGATTTCATTGAATTTGTACTCCATATTGTTTTTCC
AGATGGTATCTGGCTTGACTTTTTCTAATAAATCACCCACAACAGCCTGCCAAAGTGTTG
GAACTGCTACAGGTAGATTTTGGCCAAAACGTTTAGTTATAGTTTCCAAGGCTATTGTAG
CACCTCTTCTTTGAATTAAATTGATTTTCTGAGCATTATCTTCTTTGATAATATCCTCCA
GCTGTACCTCCATCGCTGGTGGCCTTCCTGGGCCTCTTCCACTTGATGACAGGCATCTCC
TCAGAATAGCTCTTTCTGCACTCTTGAGTTGTTTCAATAATGTTATAATAGAGGTGCGTA
AAATATCATCATCCAAAGGAACTAGGGCTTTATCATTGTTAAATGAACAGCTTTCAGCCT
TAGATGGAAAGATCCTGGGAGTAAATTCAGTGTCAGATCTAAGAAATGTGATTAGATTAG
TAATGATCTTTGCATTAGGGCAGGGTTCTCGCTCCACACAACTTTGAACCAACAAAGCTA
AATGTTTTGCAGAGAGTTCCTGCAATTCTTCATTTTCTTCACGTTTGATACTGTCCATAA
GTGGCTTTACTATTGGACTTAGCTTCTCAGGTAGTTCTTTCAGCATAACAATGGCTCCTG
CCAGTGCTGCCTGAGTCATTATATGATATGTATTCTGTTCATTGAGGGTTGTTGAAACAG
CACTACGAATACATTTCCTCCTTTCCTGTAATGTTTCTAATAACTTTGGTTTTAGTCTAC
TTTTTTCAAGCAAGTCAGCCGTATAGGTCCCACAAAGATGTTCCATTTGATCTAAAGTTA
AAACATTTGCACTGGTAATCTCATCAGCAACAACTTTGTAATGTTTCAACAAAGCAAAAA
AATCTTTGACATCTTGCAGTAATTTTGAGTAAGAGAGTCCAATCTCATCATAATAAACAG
TTTCAGTCAAGCATTCCATTAATTTGACAACAAGCGGTTTTGGAGAAGGTGATGGAAGTT
CATGCAAAGCCCATTCTGATATAACTAGACCAACAACAAACCTTTGAAGTGCAGACTTAC
TGTTCAAGTAAACTAATATCACTTTCACATAACAATCTATAGGAGACTCCATTTCCTCTG
TATAAACAATCCCAGGAGCCGGTTTCACAATGAACTTAGATAAGTATCCAAGCATTTTTG
TAACAGTAAATCTAGCATGAGAACTATTCTTTTCTCTAACATTAACAGGTACTGTCTCAT
TTCCACCAATATACTGTTTCATATCACAACGTGCTTGATCTTGTCCTTGTACACAACGGC
CGCTTTTCAAAGGACATGTTCCTTCCTTAGATGTATGTTTCGCTTGAATAAAAAGACTAG
GATCAAATGGTAAGCGGGGAGGTTGCATAGCCAAACATAGCCATCCTGTTATATAAGGAC
ACGCAGCAACTAGAATATCATTTAGCTTACTTTTGTCGAGAACTTCCTTCCAAACCACAT
GGACATTGTCATGGGTTGCCTGATGATGCTCCAGAAGAGCACGCTGATAAAGATATCTAA
GAGTCGGCTGAAGTAAAGCAGCAAAACTAGCATGTCCTGTAAGAGATGATAAAGTGTTCA
AAGCAGCAGAGCGAACACTAGTGTTATTATGTTCCAGTAAAGACCAAACTCTCATCACTA
ATTCACTTACAGCGGTATCAGCACTTATTCGAGAACTGACATTAGGTTTAAGAAGAAGTT
GAGCTAAAAGAGGTACAAAAGTGTTTGATGCTGGAGTGAGATCATCCAAGTCCGGTACTA
ACAAACATAAAGTATTGAGAACATCAACTGCTTGTTCTGGAAACTTGGAAGCCATAAGAT
CAACAGATGTAGCTAAGGCTCCAGCGGCAACTGCACCAACATCGTCCATAGCATCTTTCA
GTGATGTATGCAGTTTAGGAAAGCACAAAGGAAACAGCTCGACCAATAAGTCCTGCCTCA
CTGCAAAGATATATTTAATTCCTAGTAAGCCTCCATGCCTCGTTTCCCAGTCTTTTTGTT
CTAATAATTTCAACAAAACATTTAAAATATCATGAACACTTTGATTGCTCATTAAATTAG
CAACAGATCCTAGGACTTGAGCACAGGTTTCACGAACTGGAGCTACAACTTGGTCAAAAA
CAAAATCACCAAATCTATCTAGTGTAAG
>BMSB_*GUS*
AAAATTATAATCGGATTGTACTAGAAGATTCACATCGATTTACAAAGGAGTTTATTGTAT
AAAATATTAAAATCAATTGCCTTTTAAAATTCAATATGTTTTGACTGCTGGGACGTAACA
GGGGCAGTGTATTTGTTCAAGTCATCAGGTAAATCGAAGCCATCTAACTCATGGGCAAGC
TGCATGTATCTTTTTCTCAAATGCATAGCTGCTGCTTTTGGTTGTCGGTTTCTTGTAAAG
ACACCTTTCATGTTTCCTCCGACTCGAGTAGTGGCTTGATCAGTCTTAAAGTCTGCAAAG
TTCCAAATCATCTCACCAATGAAGAAACTATTATTTTTATTCCTAAGAGTGTCAAATGCT
TTAAAATGTTCTGAAAGCAATTCTACTTGGTACTCTTCAGACCATATGTACTCTGGCATC
AGGTGAAGTCCTGTCATCGTATCAGCACCGTATTCAGTGACAAGAACAGGTTTATTGTGC
TTTGAGTGCCACCGTTCTGCTTCTGCTTCAAGATTATATTGTATCACTTCAGTTTTTCCT
GGATATGCATACCAACCGTTATATCGATTGAAACTAAGGATGTCAACAACTTGTCCAGCA
TAGTCACTGTCTGGTCCTTGAATCATAGCTATTGTCACTGGACGCTGGACATCTAGTTGC
TTCACATGTTTTGCCACAGAGCTGAAATAATCAGCAGAAGCTGGATGTTGAGTTCTAGGT
TCGTTGGCAATACTCCATATAATAACAGACGGCCTGTTCTTATCACGTTGAATCAGCTCT
GTGAGAGAGTCTTTGTGCTTTTGAAGTAGATCTGATGAAAATAAATCTATATTGACACCA
GGGCATTCATCAATAATCATGATTCCATTTTCATCAGCAAAGTCCATGATTTCTTCAGCA
TAGGGATAATGAGATGTTCTGTATGAATTGGCTCCTATCCATTTCAAAAGGTTATAATCT
TTAGTGACTAATGGTAAATCTAATCCCCTTCCTCGAATATCTGAATCTTCATGCCTGCCA
AATCCTCTCAAATAAACAGGTTTGCCATTTATAAGGAAACTTGAATTAGTCCAGTGTATT
GTTCTTATTCCAATACGTTGCCGATAAATATCTTGTACATGATTATTCTTTTGTGTTAAA
CTTAAAAGGCGTATCTCAAGAGTATACAAGTAGCCTGGTTCTGGATTCATCATGTAAGGC

CACCATAGTTTTGCATTAGGAATCTCTGTATAACCGTTGAAACCATTTTGTTCAATTCTG
TTATTTAAGGTACTGGTTACGGGTGATCCATCAGCATCAAACAAACCTACCCAACAGCCT
ACTGAATTTATATCTTCTTTCCCACTGTAGGTGATGTTGTAGTATACAATACCTTTGGAG
CCATTTATGTCAGTATAGACAGTAACATCATCAATGTACAGATCTGGTGTAGTATATAAA
TGAACCGATCTGTGAATTCCAGCATAGTTGAAGAAATCAAATGTGTATTCCTGAACAGTT
TTTTCTCCACTTAATGTTGGTATCTTACTTAATGATCCTTGAGGCACAGTGGTAGGACTT
AGTGTATTGTCAACTGCAACAGTTATTCTGTTTTCGGTTCCATA
>BMSB_*CAT*
ATAATCACAAATCAGTATTTCTTAACACTAAGTACACTAGAAATATTGAACATTTGTAAG
TGGTTATTTTAATTCAACCTTTATTATTTAAAAATAAACTACTTTTTATTATATATTTTC
TAACTTTTTTTGATTATTCATATAGCTGAATAAGCTTGTAATTTAGCTTGAAGCATAGAT
CCAAATGTTTTATCTACTTTGAAGAAGTGACCAAGTTGTCTCTCCTGGATAAATGATGAT
GCCCTGCGAAGATGATTTGCAATATTATTAACCATTCTCTGCCTCTCATCTTCGGAAAGC
ATAGTCTGCCACATCATCGCTGGTTGGCTGTAGTTATCATCGTCTGAGGAGTCATACCTG
TCGATATCTCCAGTGGTCGATATTGGTGGAGGTCTAGCCCTCTCACTGTTGGCCATATGA
ACAAAGCTGCTTGGATAGTAGTTAGGTGCTCCATTCTGGTTGTCTCTGTAGCACATCTTA
CCGTCTCTTTGCGTATTGAATACTGGGCTTCTGAAAGGTGAATTTACTGGCAGTTGTTGA
TGATTAGGCCCAAGTCGGTGGTGGTGTGTGTCAAGGTAGGAGAACAATCGTCCCTGAAGC
ATCTTATCTGGACTTGGTTCGATTCCTGGCACCATGTTACCTGGGTTGAAGCCTGCTTGC
TCTATGTCTGTGAAATAGTTGCGGGGGTTTTTATCAAGTGTAAACCTGCCAACTTCGATC
AGAGGGAAATCGTCTTCAGGCCAAATCTTGGTTACATCGAAAGGGTTCCACCTGAACTCC
TCAGCTTCCTTGAAACTCATGACTTGGATGTAGAAGGTCCAGGAGGGGTACTTTCCCAGG
GCGATGTTGTTGTAGAGGTCTCGGATGCCATAGTCAGGATCGACTCCAGCCAACTTCTGG
GCCCTCTCCACTGGGAGGCCTCTTTCTCCTTGCGCTGTCTTGTAGTGGAATTTGCAGTAG
ACAGGTTCATTGTTCTCATTGACCAGCTTGAAGGTGTTGGAGCCATAGCCACTCATGTGC
CGGAAGCCATCGGGGATCCCTTTGTCTGAGAACAGGATCATCACCTGCATGAGGGACTCT
GGTCGCAAGCTCAAAAAGTCCCAATACATATCGTAGTCTCTCAGATGGGTGACAGGGTTC
CTCTTCTGCGAGTGGATGAATGAGAAGAAAAGAAGAGGGTCCCTCACGAAGAAGACCGGG
GTGTTGTTTCCTACCAGGTCCCATATCCCTTCATCAGTGTAGAACTTGATGGCGAACCCT
CTAGGGTCTCTGACTGTGTCTGCGCTGCCCGTTTCACCGATGACGGTAGAGAACCTGACT
AAGATGGGAGTCTTCTTGCCTACCTTTGAGAAGACCGTGGCCTTGGTGTACTGGGTGATG
TCGTTAGTGACCACGAAGTAGCCGAAGGCTCCTCCACCTTTGGCGTGTACCACCCGCTCC
GGGATCCTCTCCCTGTCGAAGTGCTGCATCTCCTCGATGAAGAAGGTGTCCTGGAGGACG
ATTGGCCCTCTAGCACCCACCGTCATGAC
